# Role of Sam68 in Post-Transcriptional Gene Regulation

**DOI:** 10.3390/ijms141223402

**Published:** 2013-11-28

**Authors:** Flora Sánchez-Jiménez, Víctor Sánchez-Margalet

**Affiliations:** Department of Medical Biochemistry and Molecular Biology and Immunology, UGC Clinical Biochemistry, Virgen Macarena University Hospital, Avenue. Sánchez Pizjuan 4, Medical School, University of Seville, Seville 41009; Spain; E-Mail: aerolazure@gmail.com

**Keywords:** Sam68, RNA-binding protein, post-transcriptional regulation

## Abstract

The STAR family of proteins links signaling pathways to various aspects of post-transcriptional regulation and processing of RNAs. Sam68 belongs to this class of heteronuclear ribonucleoprotein particle K (hnRNP K) homology (KH) single domain-containing family of RNA-binding proteins that also contains some domains predicted to bind critical components in signal transduction pathways. In response to phosphorylation and other post-transcriptional modifications, Sam68 has been shown to have the ability to link signal transduction pathways to downstream effects regulating RNA metabolism, including transcription, alternative splicing or RNA transport. In addition to its function as a docking protein in some signaling pathways, this prototypic STAR protein has been identified to have a nuclear localization and to take part in the formation of both nuclear and cytosolic multi-molecular complexes such as Sam68 nuclear bodies and stress granules. Coupling with other proteins and RNA targets, Sam68 may play a role in the regulation of differential expression and mRNA processing and translation according to internal and external signals, thus mediating important physiological functions, such as cell death, proliferation or cell differentiation.

## 1. Introduction

Increasing data has demonstrated that RNA binding proteins (RBP) and ribonucleoproteins (RNP) complexes play multiple roles in regulating a variety of biological processes by the regulation of RNA metabolism [1,2]. An emerging class of proteins participating in RNA homeostasis is represented by the Signal Transduction and Activation of RNA (STAR) family. This family includes the *Artemia salina* GRP33 [3], the *Caenorhabditis elegans* GLD-1 and ASD-2 [4], the *Drosophila melanogaster* HOW [5] and KEP1 [6], the *Xenopus* Xqua [7], QUAKING (QKI) proteins [8,9], Sam68 [10], Sam like mammalian 1 and 2 (SLM1 or KHDRBS2 and SLM2 or T-STAR, respectively) [11,12] and SF1 [13,14]. These evolutionary conserved riboproteins control a wide variety of developmental processes, integrating extracellular signals with changes in transcription and processing of target RNAs.

This family of proteins owes its name and its dual role to the presence of a structural domain for the binding of RNA, the GRP33/SAM68/GLD-1 (GSG) domain of 200 amino acids, flanked by regulatory regions containing motifs for protein–protein interactions and residues that are modified post-translationally [15]. The GSG domain contains a single hnRNP K Homology (KH) domain. KH is an evolutionarily conserved RNA binding domain that consists of 70–100 amino acids, harboring two conserved flanking sequences referred to as *N* terminus of KH (NK or Qua1) and *C* terminus of KH (Qua2) [4]. Two properties have been ascribed to this protein module: RNA-binding affinity to bipartite RNA [16–18] and the ability to homodimerize [6,16]. Moreover, some regions of the STAR proteins suggest their functional role in signal transduction as well. These sequences include proline-rich motifs, arginine glycine-rich regions and tyrosine-rich motifs in the *C*-terminal tail [13].

## 2. Sam68 Structure and Posttranscriptional Modifications

Sam68 (Src-associated in mitosis 68 kDa), also known as KHDRBS1 (KH domain containing, RNA binding, signal transduction associated 1) is the prototypic member of the STAR family of RNA-binding proteins, which regulate RNA metabolism in response to signaling cascades. Sam68 was the first STAR member to be characterized and was initially described as a cell cycle regulated phosphorylation target of c-Src and cdc2 kinases [19–21]. Moreover, Sam68 amino acid sequence and gene structure defined Sam68 subfamily of STAR proteins, as structurally different from the other two STAR subfamilies: SF1 and Quaking related proteins (reviewed in [13] and [22]).

The KH domain of Sam68 allows the binding of certain RNA sequences with high affinity and specificity. Thus, Sam68 was first shown to bind nonspecifically to poly(U) and poly(A) RNA, and specifically to the high-affinity binding sequences UAAA or UUAA *in vitro* [17,23]. 3′-UTR (3′-untranslated region) contains an AU-rich sequence, which may be considered a Sam68 target. In addition, Sam68 has been demonstrated to bind some mRNA targets *in vivo*, including beta-actin mRNA and hnRNP A2/B1 mRNA among others [24]. Other mRNA targets of Sam68 have been recently identified [25], and it remains to be determined whether Sam68 binds a rather undefined site on mRNAs or whether it requires a specific cellular context with other RNA binding proteins. Thus, while the Qua1 domain alone seems to be sufficient for dimerization of the Sam68 STAR domain, the Qua2 domain seems to contribute to the binding to some bipartite target RNAs [26]. Regarding KH function, it should be pointed out that a natural spliced variant of Sam68 lacking the KH domain was shown to be specifically expressed under arrested cellular growth, indicating that the KH domain may be necessary for the regulation of G1/S transition during the cell cycle [27].

For its role as a docking protein, Sam68 contains 5 proline-rich motifs which represent binding sites for SH3 and WW domain–containing proteins and a tyrosine rich *C*-term which can be phosphorylated and mediate SH2-dependent interplay with numerous cell signaling components in response to different stimuli [21,23]. In addition, Tyr-phosphorylation produces a negative effect on its RNA binding ability, which may be critical for the linking of extracellular stimuli to RNA processing [28]. Besides tyrosine phosphorylation, serine/threonine phosphorylation of Sam68 takes place through eight potential proline-directed MAPK phosphorylation sites [29,30]. Other posttranslational modifications have been described for Sam68: acetylation of lysine residues within the GSG domain by histone acetyltransferases positively regulates RNA binding [31], and methylation of RG (arginine/glycine)-rich regions by arginine *N*-methyltransferases is required for RNA export inducing localization to the nuclear compartment [32,33]. Also, SUMOylation (binding of a small ubiquitin-related modifier) has been described to modify Sam68 functionality by repressing the expression of cyclin D1 and resulting in G1 arrest and inhibition of cell proliferation [34].

## 3. Role of Sam68 in Signaling

Despite its predominantly nuclear localization determined by a nuclear localization signal (NLS) in the *C*-terminus [35], Sam68 was first identified as the major phosphorylation substrate for Src in the cytoplasm during mitosis [21]. In this context, not only the subcellular localization of Sam68, but also its function and affinity for RNA are strongly modulated by several signaling pathways.

Sam68 has been found to bind the SH3 domains of p85 phosphatidylinositol 3-kinase (PI3K) [19], phospholipase C gamma-1 (PLCγ-1) [23,36], protein arginine methyltransferase (PRMT) [32], Grb-2 [37], Grap [38] and Nck [39] through these polyproline motifs. Sam68 is also a target of MAPKs (via Ser/Thr phosphorylation) like Erk1/2 or Erk5 [29,40] and several tyrosine kinases such as Src, Fyn, Lck, Tec, Jak3, Brk, Zap70, Btk or the insulin receptor [41–45]. Tyrosine-phosphorylated Sam68 allows its association with numerous SH2 domain containing proteins including Src family kinases [10,21,23], Sik/BRK [41], PLCγ-1 [10], RasGAP [46,47] and Itk/Tec family kinases [48]. More recently, Sam68 has been studied to directly interact with TNFR1 (TNF-alpha receptor 1) and RIPK1 (receptor interacting protein kinase1) and also with FADD in the caspase-8/FADD/RIPK1 complex [49].

Thus, Sam68 acts as a scaffold protein in response to activation of membrane-bound receptors and extracellular signaling pathways, such as the T-cell receptor [43,48,50–52], insulin receptor [45,53], leptin receptor [54–57], TNF-alpha [58], EGF [59] or HGF/Met signaling [60]. As happens with other RBPs, the interplay between kinases, Sam68 and target RNAs have been suggested in the dynamic regulation of gene expression according to cell signaling. More precisely, a recent work has defined RNA-binding proteins, including Sam68, as a point of convergence of the PI3K and p38MAPK pathways [61]. Accordingly, Sam68 has been proposed to be a mediator of biological effects of some extracellular signals. Sam68 Tyr-phosphorylation, its negative effects on RNA binding and its participation on signaling pathways might play a key role on cellular events, such as proliferation or cell growth [62,63] providing a rapid pathway for regulating protein expression by modifying mRNA stability, processing or translation.

Regarding the biological function of Sam68, it has been considered both as a tumor suppressor and a proto-oncogene regulating cell cycle progression and apoptosis through both RNA dependent and RNA-independent mechanisms [20,27,34,64–66]. Sam68 expression level and phosphorylation status in response to extracellular stimuli play a role in tumorigenesis as well. For example, phosphorylation of the Sam68 *C*-terminal domain at Tyr440 by Breast cancer kinase (Brk) has been reported to direct Sam68 to nuclear localization and cell cycle progression [59]. Moreover, tyrosine phosphorylation of Sam68 has been shown to be elevated in human breast and prostate tumor tissues and cell lines [31,59,67], contributing to the growth, proliferation, and invasion of these cancer cells [60,68–73].

Further insights of Sam68 function and physiologic role have been provided by studying Sam68 haploinsufficiency. Even though most *Sam68*^−/−^ mice have shown normal life span and development [74], some interesting effects have been described on mice in relation to impaired fertility [75,76], delayed onset of mammary tumorigenesis and metastasis [69], basal motor coordination failure [77], maintenance of bone mass with aging [74] or protection from obesity, insulin resistance, and glucose intolerance induced with a high-fat diet by inhibiting adipogenesis [78]. These insights have also pointed out the possible role of Sam68 as a modulator of the RNA processing of some key proteins involved in signal transduction pathways, where Sam68 might have a direct participation through protein-protein interactions and posttranscriptional modifications.

## 4. Sam68 in RBP Complexes

In addition to the docking role of Sam68 in cytosolic cell signaling, Sam68 has been described to take part in some ribonucleoprotein complexes. Various other RNPs and proteins were found to be directly associated to Sam68 in the nucleus. For example, the binding to hnRNP K was described to cause antagonistic effect to their respective transcriptional activities [79]. Sam68 association to other nuclear proteins, such as hnRNP A1 [80], hnRNP G [12,81] or FAST [82] has also been described, suggesting the participation of Sam68 in pre-mRNA processing. Recently, neurological defects have been associated with alterations in hnRNPs, such as hnRNP A1 mutations, which produce familial amyotrophic lateral sclerosis [83]. Moreover, lower expression of hnRNP A/B has been found in mice models of Alzheimer disease, mediating alternative missplicing [84]. Even though Sam68 may interact with these hnRNPs, the possible role of Sam68 in these neurological alterations remains to be studied.

In cancer cells, apart from Sam68 protein showing a general nucleoplasmic distribution, Sam68 has been found to be also concentrated within subnuclear organelles called SLM/Sam68 Nuclear Bodies (SNBs), which also contain some other splicing regulators, signaling components and nucleic acids [85]. These nuclear structures show dynamic changes in response to transcriptional inhibitors, growth factors like EGF [86] and during mitosis, depending on ongoing RNA polymerase II transcription, even when these nuclear bodies do not appear to concentrate newly synthesized RNAs. Brk (PTK6), a kinase similar to c-Src, which is phosphorylated and activated by growth factor receptor signaling, was shown to colocalize with Sam68 in these dynamic spherical nuclear structures [85]. Also, Sam68-like mammalian proteins (SLM-1 and SLM-2), the heterogeneous nuclear ribonucleoproteins hnRNP A2/B1 and hnRNP G, the splicing factor YT521 [87], Sik/BRK [41] and more recently, the splicing repressor hnRNP L [88], have been described to interact with Sam68 in SNBs, thus suggesting a role for these organelles in coupling signaling to RNA processing in cancer cells.

Some cellular stresses, such as UV light, chemical insults or hyper-osmotic condition, as well as heat shock stress can induce accumulation of several RBPs in nuclear stress bodies [89]. This is a mechanism that cells adopt to limit and overcome the damage through cell cycle arrest and DNA repair. SAF-B/HAP scaffold attachment factor-B (hnRNP A1 interacting protein), ASF/SF2 and Sam68 are recruited in long tandem arrays of Satellite III (SatIII) DNA [90]. These RNP complexes are considered stress related SNBs and include other pre-mRNA processing factors that were shown to have a role in cellular response to heat shock stress [91]. Thus, these transcriptionally active granules, where Sam68 is accumulated in addition to other splicing factors, have been suggested to alter the cellular alternative splicing pattern in response to genotoxic insults, as it has been shown in tumoral cells in response to some drugs [92].

A number of different cellular structures have been demonstrated to include Sam68 in cytoplasmic localization. For instance, some other stress granules (SG), such as cytoplasmic foci, appear in cells exposed to various environmental stress agents. They are mainly composed of non-functional translation pre-initiation complexes that aggregate after phosphorylation of the alpha subunit of eukaryotic translation initiation factor 2 (eIF2α) in response to oxidative stress, hypoxia, UV exposure or heat shock [93]. Particularly, in response to oxidative stress, Sam68 was found to be recruited to SG and complexed with T-cell intracellular antigen (TIA-1), a core SG component [94], and with Ras-Gap binding protein-1 (G3BP1) [86]. The formation of viral SG in poliovirus [95] and herpes simplex virus infection [96] has been related to Sam68 as well, demonstrating the role of Sam68 in cellular responses to some viral infections. In addition, Sam68 has been reported to transiently localize in the chromatoid bodies (CB) during meiotic divisions and in early post-meiotic cells, suggesting a novel role for Sam68 in CB-linked RNA processing events and in the miRNA pathway during spermatogenesis [97].

## 5. Sam68 and Alternative Splicing

Alternative splicing (AS) is a major cause of mRNA variability and protein diversity in eukaryotes. Some sequences of RNA in the splice site or its vicinity interact with proteins that can be subdivided into SR proteins and hnRNPs [1], recruiting components of general splicing machinery or blocking this recruitment respectively. Alternative splicing is regulated in part by a network of signaling pathways which respond to extracellular stimuli [98].

Due to the binding of the splicing factor YT521-B to SAF-B to regulate the selection of alternative splice sites, and the evidence of Sam68 binding to YT521-B, Sam68 was suggested, for the first time, to be part of a signal transduction pathway that can influence splice site selection [87]. There is more evidence supporting this hypothesis, such as the described Sam68 binding to intronic regulatory pre-mRNAs [99], the binding of Sam68 to spliceosome-associated proteins [33] or to some splicing factors [12].

The molecular mechanism through which Sam68 regulates AS decisions in response to signaling cascades is poorly understood. All Sam68 and Sam68-like mammalian proteins have been implicated in alternative splicing of target pre-mRNAs [29,80,100]. Tyrosine phosphorylation of these splicing regulatory proteins seems to change splice site selection, as it was studied for p59fyn-mediated phosphorylation of SLM-1 [101]. However, insights into the biological function of these processes have only been obtained for Sam68 so far. Moreover, the role of Sam68 participating in alternative splicing has been demonstrated to be related to tumorigenesis and important cellular decisions such as cell survival or cell death [80]. Specifically, Sam68 has been shown to be frequently upregulated in some tumors, like breast cancer [102], prostate [68,103], oral tongue [72], cervical cancer [71], renal carcinoma [104] and more recently, colorectal cancer [73]. In many of them, a role for Sam68 in RNA processing and AS has been suggested.

Signaling pathways attributed to growth factors receptor activation, such as ERK1/2 pathway, seem to modulate alternative splicing through Sam68 phosphorylation, promoting some tumoral effects. CD44 encodes a cell surface molecule involved in cell adhesion, proliferation, and migration of cancer cells [105]. Ras-pathway-stimulated splicing of one variant of CD44 exons (inclusion of exon v5, which is frequent during tumor progression) in mouse T-lymphoma cells seems to be regulated by Sam68. This protein was shown to bind to sequences within the v5 exon and to be phosphorylated by ERK MAP-kinase at several sites upon Ras pathway activation [29]. Subsequent studies have shown that Sam68 cooperates with proteins that promote the inclusion of the v5 exon of CD44 alternative splicing, such as the splicing activator SRm160 [106], the chromatin remodeling protein Brm, which seems to be required for the binding of Sam68 to the RNAPII (RNA polymerase II) [107], or more recently, SND1, which also recruits Sam68 together with other factors to the pre-mRNA of CD44 in prostate cancer cells [108]. The binding of Sam68 to Brm was hypothesized to slow down the rate of RNAPII on genes regulated by the chromatin remodeling complex, thus facilitating recruitment of the splicing machinery to specific splice sites [107]. Moreover, Sam68 and Brm seem to be necessary for the alternative splicing of the human papillomavirus (HPV) polycistronic pre-mRNA, also dependent on EGF-mediated Erk1/2 activation [109]. In addition, other mechanisms have been suggested regarding how the signal-dependent modification of the Sam68 protein can affect the splicing machinery. As part of the key step in spliceosome formation, the pre-mRNA occupancy of U2AF (U2 snRNP auxiliary factor) has been shown to be downregulated by ERK-dependent Sam68 phosphorylation *in vivo*, which affects the splice site occupancy of its binding partner U2AF65 as a step to control spliceosome assembly at regulated splice sites [110].

In addition to Sam68 participation in CD44 alternative splicing correlating with enhanced malignancy and invasiveness of some tumors, this protein has been related to some other effects promoting tumorigenesis and proliferation through AS regulation. Thus, in response to extracellular stimuli, Sam68 was found to regulate the alternative splicing of the SR protein splicing factor and proto-oncogene SF2/ASF through the nonsense-mediated mRNA decay pathway (AS-NMD). Through SF2/ASF AS regulation, Sam68 has also been shown to play a role in the epithelial-to mesenchymal transition during tumor metastasis [111]. Other reported effects of Sam68 on AS in tumoral cells include the modulation of androgen receptor-dependent alternative splicing in prostate cancer LNCaP cells [103]. In these cells, also Sam68 binding to Cyclin D1 mRNA has been shown to favour the splicing of the D1b variant, which is associated with increased prostate cancer risk [112].

Moreover, Sam68 has also been shown to play a role in the regulation of Bcl-x alternative splicing in cooperation with hnRNP A1 in HEK293 cells [80]. Even though upregulation of Sam68 increases the levels of proapoptotic Bcl-x short isoform (S), Fyn-dependent Sam68 Tyr-phosphorylation seems to switch its role from proapoptotic to antiapoptotic, favoring the Bcl-x long isoform (L) splice site selection [80]. Alternatively, a similar effect was shown when the Fyn-HnRNP A2B1/Sam68-signaling pathway activation was found to reduce the proapoptotic Bcl-x(S) isoform in pancreatic cells [42]. Therefore, in addition to ERK dependent phosphorylation of Sam68, Tyr-phosphorylation was demonstrated to have a role in Sam68 modulating AS, as it was mechanistically shown by Sam68 binding to APC-arm in colorectal tumors [113]. The modulation of Sam68’s role in splicing may explain why that in cancer cells, Sam68 is distributed in the cytoplasm instead of the nucleus and is highly phosphorylated in response to some trophic and mitogenic signals [45,57]. This may lead to a differential regulation of Bcl-x splicing and combined with some other effects, may promote proliferation and metastasis.

There is in addition, mounting evidence that indicates a role of Sam68 in the regulation of alternative splicing during cellular differentiation in different tissues, namely in spermatogenesis, neurogenesis and adipogenesis.

In spermatogenesis, it has been suggested that Sam68 regulates alternative splicing at transcriptionally active sites in differentiating germ cells. Sam68 interacts with the phosphorylated form of the RNA polymerase II (RNAPII) and binds to transcriptionally active chromatin in pachytene spermatocytes, where it seems to modulate alternative splicing of mRNA targets in male germ cells, such as murine *Sgce* [100,114].

The participation of Sam68 in alternative splicing has been demonstrated also in neurogenesis, where Sam68 seems to regulate the splicing of specific pre-mRNAs, which are important for neural development [100,115]. Moreover, Sam68 has also been shown to be a key regulator of activity dependent alternative splicing in the central nervous system [116]. Besides, the splicing activity of Sam68 has recently been implicated in the onset of two human neurodegenerative diseases. Thus, recent studies have found Sam68 colocalized with MBNL1 (muscle blind-like), and hnRPN G proteins within CGG mRNA aggregates, which are supposed to play a role in the regulation of AS in Fragile X-associated tremor/ataxia syndrome (FXTAS). This neurodegenerative disorder is due to an RNA gain-of-function mechanism, where RNA toxicity due to the elevated FMR1 mRNA levels is observed in premutation carriers. Sam68 is sequestered by expanded CGG repeats and thereby loses its splicing-regulatory function whereas Sam68 Tyr-phosphorylation seems to reduce its recruitment [117]. In the neurodegenerative disorder spinal muscular atrophy (SMA), Sam68 has been shown to be a novel and crucial regulator of SMN2 alternative splicing, acting as a splicing repressor of exon 7 inclusion through both its cooperation with hnRNP A1 and its RNA binding ability [118,119].

More recently, Sam68 has been reported to regulate alternative splicing of the PI3K downstream effector mTOR in adipogenesis. Sam68 deficiency resulted in mTOR intron 5-retention through the introduction of a premature termination codon and the subsequent reduction of protein levels of mTOR [78]. Thus, Sam68 activation and participation in the PI3K pathway, as previously reported [45,62], may also mediate the regulation of the abundance of PI3K downstream kinases, such as mTOR. Along these lines, other studies have linked RNA-binding proteins participation in PI3K and MAPK signaling pathways by phosphorylation [61].

## 6. Other Sam68 Functions in RNA Metabolism: Transcription, Translation, miRNA Processing and RNA Transport

Sam68 could also have a role in transcriptional regulation and may behave as a competitive inhibitor of positive regulators of transcription, as has been shown by repressing various mammalian and viral promoter constructs. Thus, binding of Sam68 to hnRNP K inhibits the function of hnRNP K in transcriptional activation of a reporter driven by the CT promoter element of the proto-oncogene c-myc [79]. Most of these functions of Sam68 regulating transcription are mediated by protein-protein interaction, as previously shown with the inhibition of the transcriptional activity of the multifunctional adaptor CBP [120]. In this context, there is increasing evidence for the concept of spatial and temporal coupling between splicing and transcription, especially with nuclear factors [121]. Sam68 also directly interacts with the androgen receptor and binds to androgen responsive elements (AREs) within the promoter region of the *prostate-specific antigen* (*PSA*) gene, where Sam68 seems to have some effect on AR-regulated transcriptional activity independently of its ARN binding capacity and splicing regulatory properties in LNcaP cells [103]. Sam68 may also be acting as a co-activator of ER-dependent transcription in mammary development and tumorigenesis [69]. Moreover, Sam68 is required to guarantee proper expression of the gonadotropin receptor transcripts in pre-ovulatory follicles from adult ovary with a possible role upregulating both the FSH and LH receptor transcripts [75].

In addition to transcriptional and posttranscriptional functions of Sam68, increasing evidence has shown that Sam68 could also play a role in translation [122]. In male germ cells Sam68 is mainly nuclear, but it translocates into the cytoplasm of secondary spermatocytes, where it regulates the translation of specific mRNA targets in association with polysomes [30,76]. In secondary spermatocytes and early round spermatids, Sam68 has been shown to interact with the translational machinery, associating to eIF4F in an effect that seems to be strongly correlated with ERK-dependent Sam68 phosphorylation [76].

Recent studies have also proposed a role for Sam68 in the miRNA pathway during its translocation and accumulation in chromatoid bodies in spermatogenesis [97]. Based on previously known Sam68 interaction with DROSHA and DICER, the two RNase III enzymes involved in the nuclear and cytoplasmic processing of the miRNA precursors [123], twelve miRNAs were differentially shown to be expressed between wild-type and knockout Sam68^−/−^ germ cells. These miRNAs included miR-29b, which belongs to a family of miRNAs that are often down-regulated in human cancers [124]. Thus, increased expression of Sam68 in some cancer cells, such as prostate cancer, may be required to suppress miR-29b expression and promote an aggressive phenotype [111,125].

Most of the evidence regarding the role of Sam68 in RNA export has been based on studies of retroviral mRNA processing [126]. The human immunodeficiency virus (HIV-1) differentially controls viral protein expression at the level of splicing as well as nuclear export of incompletely spliced viral RNA, mediated by Rev protein. Sam68 was initially found to bind to Rev and synergize not only CRM1-dependent RNA transport but also RRE-mediated gene expression and viral replication of HIV-1 [127,128]. Direct evidence of the essential role of Sam68 in nuclear export of RRE-containing RNA and Rev function was shown by reducing Sam68 expression, which caused dramatic inhibition of HIV-1 production [129]. Also, Sam68 tyrosine phosphorylation was observed to impair Sam68 ability to facilitate the export of unspliced RNAs [41]. In other contexts, Sam68 may promote the transport and association of mRNAs to specific cellular localizations. Recently, Sam68 has been shown to promote the association of Beta-actin mRNA with synaptic polyribosomes specifically in the synaptodendritic compartment, thus regulating hippocampal synapse number [130].

## 7. Conclusions

Sam68 has been suggested to participate in a variety of biological processes regarding its apparent multifunctionality as a prototypic member of STAR family of proteins. Sam68 structure justifies its docking function in signaling, where SH3 and SH2 interactions, in addition to Sam68 phosphorylation, mediate signal transduction of extracellular stimuli.

Sam68 posttranscriptional modifications, its association to other proteins and RNA targets seem to account for both Sam68 function and its intracellular dynamics. Thus, Sam68 shows different biological functions depending on the signaling status of the cell, which may mediate a role of Sam68 in transcriptional processes, posttranscriptional events, such as alternative splicing, regulation of miRNA pathway, translation, or RNA transport. Nevertheless, further studies are needed to understand the specific role of Sam68 integrating the broad variety and complexity of external and internal signals in relation to its participation in multi-molecular complexes and the modulation of key steps of RNA processing. More precisely, a better knowledge of the contextual interaction of many proteins and RNA molecules that may associate with Sam68 is needed. Moreover, further studies investigating the biological effects of Sam68 downregulation or knockout may be useful to clarify the role of Sam68 in the processing of specific RNA targets and how it may affect biological processes. Finally, regarding Sam68 biological functions and its implication in some pathophysiological conditions (see Table 1), further studies combining basic and clinical approaches may be necessary to confirm the relevance of Sam68, as well as to define its putative use, both as a molecular marker and as a molecular target, for the development of new therapies.

## Figures and Tables

**Table 1.** Sam68 participation in different diseases.

Disease	Effect	Role of Sam68	Suggested mechanism	Ref.
Fragile X-associated tremor/ataxia syndrome (FXTAS)	Clinical disease	Regulation of alternative splicing	CGG repeats recruit Sam68	[117]

Spinal muscular atrophy (SMA)	Clinical disease	Regulation of alternative splicing	Sam68 is repressor of exon 7 inclusion of SMN2	[118]

Breast cancer	Tumor progression, tumorigenesis, metastasis	-Sam68 overexpression and cytoplasmic localization -Sam68 haploinsuficiency delays onset of mammary tumorigenesis and metastasis	-Complex formation (Brk, ERK5, Sam68) under MET receptor activation	[60]
			-Cell cycle regulation	[102]
			-Sam68 modulation of Tyr kinase activity	[69]

Prostatic cancer	Neoplasmic transformation of prostatic cells	-Src depending Sam68 phosphorylation -Sam68 overexpression	-Nonregulated Sam68 phosphorylation may alter truncated c-kit expression	[67]
			-Cell cycle regulation	[68]

Colorectal cancer	Tumor progression	Sam68 overexpression and nuclear localization	Unknown	[73]

Cervical cancer	Tumor progression	Sam68 overexpression and cytoplasmic localization	Regulation of epithelial/mesenquimal transition	[71]

Renal cell carcinoma	Tumor progression	Sam68 overexpression and cytoplasmic localization	Unknown	[104]

N0 oral tongue cancer	Tumor progression	Sam68 overexpression and cytoplasmic localization	Unknown	[72]

Infertility/Subfertility	Alteration of ovary function and spermatogenesis defects	-Disregulation of RNA metabolism in *Sam68* knockout mice	-Binding/downregulation of FSH and LH receptors mRNAs	[75]
		-Regulation of protein translation	-Interaction with translational machinery in polysomes.	[76]
